# Moving towards a more inclusive patient and public involvement in health research paradigm: the incorporation of a trauma-informed intersectional analysis

**DOI:** 10.1186/s12913-017-2463-1

**Published:** 2017-08-07

**Authors:** Carolyn Shimmin, Kristy D. M. Wittmeier, Josée G. Lavoie, Evan D. Wicklund, Kathryn M. Sibley

**Affiliations:** 1 Centre for Healthcare Innovation, 753 McDermot Ave, Winnipeg, MB R3E 0T6 Canada; 2 Department of Pediatrics, University of Manitoba, 375-753 McDermot Ave, Winnipeg, MB R3E 0T6 Canada; 3 University of Manitoba, Faculty of Community Health Sciences, 379-753 McDermot Ave, Winnipeg, MB R3E 0T6 Canada; 4 Ongomiizwin- Research, Indigenous Institute of Health and Healing, 715 John Buhler Research Centre-727 McDermot Ave, Winnipeg, MB R3E 3P5 Canada; 5 Department of Disability Studies, University of Winnipeg, 515 Portage Avenue, Winnipeg, MB R3B 2E9 Canada; 6 Canadian Centre on Disability Studies, 226 Osborne Street North, Winnipeg, MB R3J 1T2 Canada

## Abstract

**Background:**

The concept of patient engagement in health research has received growing international recognition over recent years. Yet despite some critical advancements, we argue that the concept remains problematic as it negates the very real complexities and context of people’s lives. Though patient engagement conceptually begins to disrupt the identity of “researcher,” and complicate our assumptions and understandings around expertise and knowledge, it continues to essentialize the identity of “patient” as a homogenous group, denying the reality that individuals’ economic, political, cultural, subjective and experiential lives intersect in intricate and multifarious ways.

**Discussion:**

Patient engagement approaches that do not consider the simultaneous interactions between different social categories (e.g. race, ethnicity, Indigeneity, gender, class, sexuality, geography, age, ability, immigration status, religion) that make up social identity, as well as the impact of systems and processes of oppression and domination (e.g. racism, colonialism, classism, sexism, ableism, homophobia) exclude the involvement of individuals who often carry the greatest burden of illness — the very voices traditionally less heard in health research. We contend that in order to be a more inclusive and meaningful approach that does not simply reiterate existing health inequities, it is important to reconceptualize patient engagement through a health equity and social justice lens by incorporating a trauma-informed intersectional analysis.

**Summary:**

This article provides key concepts to the incorporation of a trauma-informed intersectional analysis and important questions to consider when developing a patient engagement strategy in health research training, practice and evaluation. In redefining the identity of both “patient” and “researcher,” spaces and opportunities to resist and renegotiate power within the intersubjective relations can be recognized and addressed, in turn helping to build trust, transparency and resiliency — integral to the advancement of the science of patient engagement in health research.

## Background

### The concept of patient engagement

The concept of patient engagement in health research — defined as the meaningful and active involvement of patients in: the governance; priority setting; conducting; and translation of research [1] — has received growing international recognition over recent years. Patient engagement in health research is driven by the belief that greater involvement of patients throughout the research process (including as co-researchers) will lead to improved outcomes and an enhanced healthcare system, and will improve the quality, appropriateness, acceptability, transparency and relevance of research — ensuring it addresses issues of importance to patients and the public [2–9].

But the concept of patient engagement is problematic as it negates the very real complexities and context of peoples’ lives. Though it conceptually begins to disrupt the identity of “researcher,” and complicate our assumptions around expertise and knowledge, it continues to essentialize the identity of “patient.” When approaches and methodologies are created based upon a definition of “patients” as a homogenous group who need to simply be more “engaged” by health researchers it denies the reality that individuals’ economic, political, cultural, subjective and experiential lives intersect in intricate and multifarious ways. It also excludes the involvement of individuals who may not identify as “patients” for a myriad of reasons — people who are unable to access the healthcare system because of geography and/or systemic barriers (including racism, colonialism, sexism, classism, ableism and heterosexism); individuals living with mental health or substance use issues who may be hesitant in taking up the identity of patient due to the associated stigma attached to such a label; and people who refuse to engage or prematurely exit the healthcare system because of unresponsive or disrespectful care. Also of importance around the essentialization and categorization of patients, are individuals living with the label of intellectual and/or physical disability who have experienced continued medicalization and objectification (e.g. disability conceptualized as something in need of fixing or to be overcome) as a means to regulate and discipline non-conformist bodies (i.e. objectified by the medical gaze, the oppression then internalized and made manifest through self-regulation and policing derived from feelings of shame and guilt) [10, 11].

### A social gradient in health

Substantial and robust evidence points to the existence of a social gradient in health, meaning social and economic conditions and their effects on people’s lives determine: their risk of illness; the actions they are able to take in order to prevent themselves from becoming ill; and treating illness when it does occur [12–14]. For example, cardiovascular disease mortality in Canada is highest among those in the poorest income group and as income increases, mortality rate decreases [15–17].

It is also important to consider what is known as the inverse care law — the principle that the availability of good medical or social care tends to vary inversely with the needs of the populations served [18]. This is based on the theory that market forces influence the distribution of services more strongly than population need. For example, in Canada obstetrical services are provided where they are most efficient and profitable rather than on the basis of need alone – so optimal allocation of limited obstetrical services in Canada dictates that services are concentrated in areas with the largest populations (i.e. large urban centres). This may disadvantage women living in rural and remote areas as well as their families [19]. A study on maternal health [20] discussed the problematic nature of allowing childbirth research priorities to be determined by the vociferous and privileged families living in large urban centres that may end up diverting crucial limited resources away from areas of greater clinical need – pointing to the need for inclusive practices in engagement that involves individuals who may face a number of barriers to access to healthcare, including geographical and systemic.

The social gradient to health as well as the inverse care law make evident that patient engagement strategies and approaches that do not consider the simultaneous interactions between different social categories (e.g. race, ethnicity, Indigeneity, gender, class, sexuality, geography, age, ability, immigration status, religion) that make up social identity, as well as the impact of systems and processes of oppression and domination (e.g. racism, colonialism, classism, sexism, ableism, homophobia), risk excluding the involvement of individuals who often carry the greatest burden of illness — the very voices traditionally less heard in health research.

### Reframing the central goal of patient engagement

The roots of patient engagement originate from the world of HIV/AIDS research, [21] as well as the disability movement that coined the phrase, ‘Nothing about us without us’. It grew out of a sense of democratic purpose – that people who are affected by research have a right to have a say in what and how publicly funded research is carried out. But there has been a shift in recent years to conceptualizing the involvement of patients in health research as either a means towards better personal health decision-making (i.e. the involvement of the public will increase their understanding of health research which in turn will strengthen their ability to make good health decisions for themselves and their families) [22] and/or toward enhancing research (i.e. the involvement of patients in health research will improve the quality, transparency, relevance and accountability of research) [23]. This change in thought and conceptualization risks a top-down approach that centres on the primacy of researchers’ needs over the needs of communities; in turn replicating, reproducing and reconstituting already existing inequities by privileging certain voices — mainly white, middle-class people who: have relatively fair access to health care; can navigate the system with comparative ease; and feel comfortable with identifying as a patient — over others [24].

But what if we were to reframe the central goal of patient engagement in health research as a matter of health equity and social justice? Similar to community participatory research approaches, [25] used mainly in the social sciences, only further expanding where these participatory frameworks often leave off by addressing the impact of the complex interplay between systems of power and formation of identity of both researchers and patient co-researchers. And instead of the term patient engagement we would encourage the use of the term public involvement (often used in the UK and Australia with regard to engagement in health research activities) or the involvement of people with lived experience, in order to highlight the need for a variety of voices and perspectives in health research. Also instead of patient co-researcher, we would be in favour of using the term public research partner which helps to underscore the partnership and relationship-building aspect necessary in any engagement approach. We argue that this would in turn: firstly, expose spaces and opportunities for potential resistance and resilience when it comes to intersubjective power relations effectively present in all engagement activities; secondly, reveal the truly transformative nature of collaboration in health research; and thirdly, aid to improve health outcomes of ALL while also paying attention to the reduction of health inequities.

Therefore, if one of the primary principles behind patient engagement in health research is inclusivity — and the key driver is to improve health outcomes and enhance the healthcare system — we would argue that a public involvement in health research strategy must incorporate a trauma-informed intersectional analysis. This will ensure that health inequities are not simply reiterated, reconstituted and reproduced through involvement approaches and methodologies that are meant to make certain that issues of importance to those living with complex health needs — and this includes the social determinants of health — are being addressed.

### What is an intersectional analysis?

Historically speaking, the concept of intersectionality emerged from a myriad of theoretical groundings including: US Black feminism; Indigenous feminism; third world feminism; and queer, poststructuralist and postcolonial feminism [26–33]. The term itself was first officially coined by Kimberlé Crenshaw [26] in 1989. Intersectionality promotes an understanding of human beings as shaped by the interactions of different social locations or categories ― for example, race, ethnicity, Indigeneity, gender, class, sexuality, geography, age, ability, migration status, and religion. It is important to remember that from an intersectional perspective, social categories are considered: dynamic; historically grounded; socially constructed; and working on both the micro and macro structural levels [34, 35]. Interactions between social categories occur within a larger context of connected systems and structures of power (e.g. laws, policies, governments, media, and public institutions). Through these processes, interdependent forms of privilege and oppression shaped by colonialism, imperialism, racism, homophobia, ableism and patriarchy are created. To put it more simply, prominent intersectional theorist, Olena Hankivsky [36] writes,

*According to an intersectionality perspective, inequities are never the result of single, distinct factors. Rather they are the outcome of intersections of different social locations, power relations and experiences.* (p.2)

A central goal of intersectionality is the social inclusion of voices traditionally less heard, including populations often ignored, excluded and/or objectified — which is also key to the concept of public involvement in health research [37]. This is achieved by various means including conceptualizing social categories as interacting with and co-constituting one another to create unique social locations that vary according to time and place (this is often visualized as an oscillating web, where there are spaces and opportunities for renegotiation and resistance to power) [38]. Intersectionality involves a multi-level analysis that endeavours to understand the effects *between and across* various levels in society including: macro (i.e. global and national-level institutions and policies); meso (i.e. provincial and regional-level institutions and policies); and micro levels (i.e. community-level, grassroots institutions and policies as well as the individual or ‘self’). It focuses on the intersecting processes by which power and inequity are produced, reproduced and actively resisted across levels of structure, identity and representation [39, 40].

### The role of trauma in health

An important but often overlooked aspect in the practice of public involvement in health research is the recognition that the experiential knowledge of the public often sought out by researchers in involvement activities may be intertwined with experiences of trauma. For example, evidence shows that many patients in primary care settings have significant trauma histories which have an impact not only on their health but also on their responsiveness to health interventions [41–43]. Experiences of trauma may also impact an individual’s ability to access appropriate healthcare services and hence to identify as a patient.

Embedded in any public involvement in health research practice must be the recognition that trauma is a widespread, harmful and costly public health issue. It occurs as a result of violence, abuse, neglect, loss, disaster, war and other emotionally harmful experiences. Trauma is often seen as an almost universal experience of people living with mental health or substance use issues [44].

Moreover, there needs to be an acknowledgement that healthcare systems that are intended to provide services and supports to individuals may be trauma-inducing. For example, the use of coercive practices, such as seclusion or restraints, in the behavioural health system or the use of invasive procedures in the medical health system can be re-traumatizing to individuals who may have already experienced significant histories of trauma before entering the system. The same can be found in areas such as dental health, where individuals who have been victims of torture and may have experienced significant trauma to the mouth — including First Nations peoples in Canada who received dental care without anesthetics in the 1960s — may require special additional supports due to fear of contact by those in the health care profession that they do not know.

The pervasive and harmful impact of traumatic events on individuals, families, caregivers and communities and the unintended but similarly widespread re-traumatizing of individuals within our public institutions and service systems — not only healthcare systems but also education systems, corrections systems, child welfare systems, government interactions, etc. — makes it necessary for any public involvement in health research practice to involve a trauma-informed approach.

Once more, there needs to be an understanding that trauma does not live solely in the realm of the public research partner who is being asked to share their experiential knowledge but also for the researcher themselves. Evidence shows that in some instances, past experiences of trauma may indeed be a driving motivator for certain researchers in the work they do, exemplified by the wounded healer paradigm [45] often discussed in the field of behavioural health sciences. In addition, even in areas of research where the researcher may not have directly experienced the health conditions being investigated, for example in the field of gerontology, stories shared by public research partners may in time be researchers’ own [46]. It is a reminder that researchers may have a personal connection with hopes and fears expressed by public research partners, and that stories of abuse, loneliness, racism, sexism, etc. do not occur in a vacuum and are likely to have an impact on both researchers and public research partners.

Traumatic events by their very nature set up a power differential where one entity (whether an individual, an event, a system or a force of nature) has power over another. An individual’s experience of these events or circumstances are shaped in the context of this powerlessness and feelings of humiliation, guilt, shame, betrayal or silencing often shape the experience of this event. It is important that in interpersonal interactions — something that plays a very large role when it comes to public involvement in health research — that these feelings of powerlessness are not reproduced or reconstituted in any way.

### A trauma-informed approach

According to the U.S. Department of Health and Human Services Substance Abuse and Mental Health Services Administration’s guidelines [47] a trauma-informed approach includes: creating methodologies that recognize the widespread impact of trauma; understanding potential paths of recovery; recognizing signs and symptoms of trauma; and seeking to actively resist re-traumatization through the creation of both physical settings and interpersonal processes that support safety for both researchers and public research partners [47–49].

By ensuring that an intersectional analysis within a public involvement in health research framework is trauma-informed, both researcher and person with lived experience may find common ground and the process of ‘othering’ (i.e. “researcher” vs. “public research partner”), that may ostensibly limit the inclusivity of current engagement approaches, is disrupted. This means the instability of the binary categorization of “patient” or “public” and “researcher” is exposed, in turn revealing spaces where power can be resisted and renegotiated ― which not only works to strengthen trust within the researcher/public research partner relationship but also helps to build resiliency on both sides.

### An indigenous intersectional analysis

In Canada, Australia, New Zealand and the United States, where governments inspired by settler societies dominate, it is important to foreground anti-colonialism and the varieties of Indigenous sovereignty/nationhood aspirations when incorporating a trauma-informed intersectional analysis in public involvement in health research. This involves an examination of the role of colonization, both past and present, in violence against Indigenous peoples. For example, in Canada this would involve consideration of the legacy of the residential schooling system (from the 19th century until 1996, around 150,000 First Nation, Inuit and Metis children were removed from their families and communities and forced to attend residential schools where they were subjected to physical, spiritual, emotional, psychological and sexual abuse) and the 60s scoop (where from 1960 to 1980 thousands of Indigenous children were taken from their families, often without their parent’s knowledge or consent, and fostered or adopted out to primarily white, middle-class families within Canada as well as the United States and Western Europe) within the child welfare system. Clark [50] writes that an Indigenous intersectional analysis should include: 1) an analysis of policy and policy intersections as colonial violence; 2) an anti-colonial gender analysis; 3) a contextualization of individuals within community and family histories; 4) a positioning of agency as central; and 5) an acknowledgement of resistance.

This expanded intersectional analysis is particularly important in a public involvement in health strategy that is seen through a health equity and social justice lens. For example, if we were to look at the intersections of colonialism, racism and sexism in Canada, research demonstrates that Indigenous women in Canada carry a disproportionate burden of ill health and disease, including higher rates of heart disease, hypertension, diabetes, cervical and gallbladder cancer, HIV/AIDS, substance use problems, mental illness and suicide [51–56]. Evidence shows First Nation women are more likely to die of a treatable or preventable illness [57].

Adding a trauma-informed approach to this analysis, we would also recognize that the overrepresentation of Indigenous children in the child welfare system is not a remnant of the past, but rather a fact that remains an urgent and ongoing challenge facing Indigenous communities across Canada [58]. While the most recent Statistics Canada population estimates suggest that Indigenous people account for slightly more than 4% of the general Canadian population, Indigenous children represent 48% of children in care [59]. In a recent population-based study in Hamilton, Ontario, it was found that of First Nations adults who had experienced involvement of a child protection agency in their own personal care as a child, 49% felt that it had a negative effect on their overall health and well-being [60]. Participants in this study also identified dislocation from traditional lands (29%) and residential school attendance by a family member (34%) as having negative impacts on their health and well-being [60]. The study also found a population prevalence of post-traumatic stress disorder of 34% [61]. This points to the importance of foregrounding anti-colonialism and Indigenous knowledges when incorporating a trauma-informed intersectional analysis in public involvement in health research strategies.

## Discussion

### How a trauma-informed intersectional analysis can inform public involvement in health research strategies

In acknowledging: firstly, the problematic nature of the conceptualization of patient engagement especially with regard to the inclusion of voices traditionally less heard in health research; and secondly how the incorporation of a trauma-informed intersectional analysis might lead to more authentic and meaningful involvement of people with lived experience, their families and communities in health research; the question then becomes how do we bridge the theory to action gap in order to ameliorate engagement practice? We propose a number of ways below in which a trauma-informed intersectional analysis can be integrated into training, practice and evaluation of public involvement in health research. We recognize that the incorporation of a trauma-informed intersectional analysis may be a challenge for some health researchers — this is why you might notice that the tables of questions and considerations in each section below build upon each other, allowing research teams to work at their own pace and level of comfort, gradually implementing over time depending on the phase and stage of the research process. Also of note, for health researchers engaging the general public, the following tables can be easily adapted to the context, allowing for the incorporation of a trauma-informed intersectional analysis in all public participatory approaches.

## Building capacity: learning reflexivity

### Discursive reflexivity

An important component of an intersectional analysis is the exploration of power. One way intersectionality pays attention to power is through discursive reflexive practice. Reflexivity acknowledges the importance of power at the micro level of the self and our relationships with others, as well as the macro levels of society. It recognizes the multiple truths and a diversity of perspectives, while giving extra space to voices typically excluded from ‘expert’ roles [62].

For researchers, reflexivity is an important practice skill that is central to working ethically in uncertain contexts and unpredictable situations, which can often be the case in the development and building of public research partnerships. Practicing reflexivity requires both researchers and public research partners to commit to ongoing dialogues about implicit personal and professional knowledges and the construction of expertise in academia [63]. It exposes how researchers’ assumptions about social, economic and health problems, and the people who experience these problems, have ethical and practical consequences.

Reflexivity can help to transform the process of public involvement in health research when both researchers and public research partners who are being engaged bring critical self-awareness about the assumptions and ‘truths’ in their work [50]. An example of this includes reflexive practice to help people consider their individual connections to colonization which then helps to facilitate questioning around policy, practices and research (both past and present) that are used in the colonization of Indigenous peoples in Canada [64].

A comprehensive public involvement in health research training for both researchers and public research partners must include teachings around discursive reflexive practice. Adapting from Olena Hankivsky’s *Intersectionality-Based Policy Framework* [65] as well as *SAMSHA’s Guidance for a Trauma-Informed Approach* [47] the following types of questions should be considered in public involvement in health research (Table 1).

**Table 1:** Discursive reflection

Questions for research project team (both Researchers and Public Research Partners)
1. What are my own personal values, experiences, interests, beliefs and political commitments in the area of health we will be researching?
2. How do these personal experiences relate to social and structural locations (e.g. gender identity, race, ethnicity, Indigeneity, socioeconomic status, sexuality, gender expression, age, sexual orientation, immigrant status, religion) and processes of oppression (e.g. patriarchy, colonialism, capitalism, racism, heterosexism, ableism) in the area of health in which we will be researching?
3. What are my personal values, assumptions, perspectives and experiences with regard to people living with the health condition(s) or issue(s) in which we will be researching?
4. From your perspective, what current health inequities (i.e. avoidable and unjust inequalities in health between and within groups of people) exist with regard to the area of health in which we will be researching?
5. How do you think people with lived experience in this area of health would prefer to be involved in research and why? What types of challenges do you think would need to be addressed in order to make it easier for people living with this health condition or issue, as well as their families and communities to become involved in research?
6. Working together, how can we become more aware of and take advantage of opportunities where we can challenge each other’s ideas and renegotiate power within our project team? What does building resilience look like, feel like, and sound like to you?
7. How do you think the issue of trauma may impact the area of health in which we will be researching? (Remember to think about it both on the level of violence within relationships but also on the larger level of colonialism, racism, sexism, homophobia, capitalism, ableism, etc.)
8. What do you think are some of the ways in which we can make sure everyone feels safe when working together on this research project? What does physical safety mean to you? Look like to you? Feel like to you? What does emotional/psychological safety mean to you? Look like to you? Feel like to you? What are some of the best ways we can work together to address trauma? (This will be discussed as well in the practice section)

### Embodied reflexivity

Any public involvement in health research training that teaches discursive reflexive practice should also take one step further than what is typically involved in an intersectional analysis and include embodied reflexive practice ― looking at how a researcher’s physical presence and performance potentially influences the public research partner’s sharing of their experiential knowledge. This means exploring the role of embodiment in: the construction of particular narratives; the production of knowledge; and specific types of research relationships.

Oftentimes public involvement in health research activities are predicated on the public research partner’s sharing of their personal physical interaction with their environments. There is sometimes a juxtaposition created between the culturally-constructed vulnerabilities of what may be falsely deemed as the ‘failing body’ of the public research partner and the researcher’s sometimes seemingly ‘vibrant physicality’ [66]. A researcher’s insight and awareness into their own physical capabilities and how they interact with the environment could in turn make a critical impact in the engagement process. It also helps to simultaneously challenge, disrupt and trouble the socially-constructed discursive practice of binary categorization of what is deemed as worthy vs. unworthy bodies [67].

What is enhanced by engaging the embodied researcher and public research partner is the possible opportunities present in the intersubjective space within the partnership. The current state of patient engagement in health research neglects the exploration of intersubjective space within difference, even though difference is inevitable and social and political histories such as colonization, migration and residence status — related to power and potential language differences — can create issues that cannot simply be ignored, and must surely be methodologically explored and exposed both to the challenges as well as the possibilities they create.

One of the most important things in discursive and embodied reflexive practice is how it challenges the simple and static constructions of ‘self’ and ‘other’ [68] — which current patient engagement practices tend to avoid, maintaining the differential positioning of researcher and public research partner within the process of research knowledge and production. But in having both researcher and public research partner trained in discursive and embodied reflexive practice before engaging on a research project together, may expose the complex interplay that belies divergent constructions of research roles and personal/social identities that occur during public involvement practice in which multiple identities are performed, negotiated and reconstructed. In precluding the essentialization of researcher and public research partner roles and responsibilities through reflexive practices, we open up spaces where new roles and responsibilities in the production of research knowledge can be explored.

## Enhancing the practice: disrupting the status quo

It is important that approaches to public involvement in health research do not inadvertently disadvantage or harm any particular individual or community or, on the other hand, be complicit in the empowerment of another. Integral to the utilization of a trauma-informed intersectional analysis in the development of inclusive public involvement approaches is the examination of how subjects construct, develop, and negotiate their own social locations and those of others in social contexts of power. Dhamoon [69] looks at the representation of different dimensions of socio-political life as a four-pronged endeavour, examining: 1) identities of individuals or social groups; 2) categories of difference; 3) systems of domination; and 4) processes of subject formation. Adapting from Olena Hankivsky’s *Intersectionality-Based Policy Framework* [65] as well as *SAMSHA’s Guidance for a Trauma-Informed Approach* [47] the following types of questions should be considered before deciding on a public involvement approach in the context of health research (Tables 2, 3 and 4).

**Table 2:** Framing and prioritizing the research question

Questions for the research team (i.e. Researchers and Public Research Partners)
1. What is the research problem we will be looking at? ➢ How did we decide upon this research problem? ➢ Did we involve people/families/communities with lived experience of this health issue in prioritizing this research problem? ➢ Is there anyone we have not involved who should be?
2. What assumptions (e.g. beliefs about what causes the health issue we are researching and which population(s) is/are most affected) do you think underlie the representation of this health issue we will be looking at? ➢ Who do you think is involved in framing and defining the health issue in that way? ➢ Why do you think they frame the health issue that way? ➢ Do you think that the way people look at the health issue has changed over time (i.e. historically), or is different depending on where they live (i.e. geographically)?

**Table 3:** Understanding the different populations affected

Questions for the research team (i.e. Researchers and Public Research Partners)
1. What inequities (i.e. avoidable and unjust inequalities between and within groups of people) exist in relation to the health issue we will be researching? (Remember to think about *intersecting social and structural locations* such as gender identity, race, ethnicity, Indigeneity, socioeconomic status, sexuality, gender expression, sexual orientation, immigrant status, religion as well as *processes* such as patriarchy, colonialism, capitalism, racism, ableism and heterosexism)
2. Where should we look to find the necessary information to help us answer this research question?
3. In what ways do you think we could get a conversation going about the health issue we are researching across different groups of people who may be differently affected by the same health issue?
4. When thinking of the different groups of people who may be differently affected by the same health condition or issue, what do you think are things that we still need to work at better understanding? (i.e. knowledge/evidence gaps)

**Table 4:** Deciding on the engagement strategy

Questions for the research team (i.e. Researchers and Public Research Partners)
1. What role(s) do you think people with lived experience, their families and communities could play and would like to play in conducting this research?
2. How do you think we can make sure that everyone’s perspectives are included, and that we address inequities (i.e. avoidable and unjust inequalities between and within groups of people) as well as issues of social justice (i.e. justice in terms of the distribution of wealth, opportunities and privileges within society)?
3. How can we make sure when we come together that we do not reinforce existing stereotypes or biases or produce further inequities (i.e. avoidable and unjust inequalities) for some people and populations?
4. What do you think would be the best way for people with lived experience, their families and communities to be involved in making sure that the outcomes or results of the research lead to a reduction in inequities (i.e. avoidable inequalities between and within groups of people)?
5. In what ways do you think we can work together to make sure everyone on the research team as well as any people involved in the research project feels safe?
6. How do we make sure that the physical setting for engagement activities is considered safe by ALL members of the research team (as well as any participants of the research study)? What makes you feel physically safe? What types of things should we think about when we are meeting to ensure our environment and physical space is considered safe by everyone?
7. How do we make sure that interpersonal interactions promote a sense of safety for ALL members of the research team (as well as any participants in the research study)? What makes me feel psychologically safe? What types of interactions do not make me feel safe and should be avoided?

It is integral that in any engagement approach that there is an ongoing dialogue around the dynamic nature of systems of power and their effects upon the partnership between researchers and public research partners in order to continually reassess and expose where there may be spaces in intersubjective relations that allow for opportunities for resistance and renegotiation of power and hence transformative collaboration.

## Thinking about evaluation: measuring success from a health equity and social justice lens

Though the evidence base demonstrating that the perspective of people with lived experience plays an integral role in the process by which research is identified, prioritized, designed, conducted and disseminated, continues to grow ― evidence of the relative impact of public involvement in health research is still limited and weak at this time, primarily due to poor reporting, with many studies only providing partial information and a lack of consistency in terminology [2, 70]. This in turn can impact our overall understanding of public involvement in health research ― of what works, for whom, in what context and why [9].

But this also offers an opportunity, not only to improve upon the conceptualization and interpretation of what is public involvement in health research ― of how theory meets practice (as we have done above) ― but also to set evaluative measures that will ensure that the involvement of people with lived experience leads to improved outcomes for ALL in health research and addresses issues of health equity and social justice. Evaluative measures have to not only look at the level of public engagement in health research (whether it be information sharing, consultation, collaboration or community-driven) but also the quality and integrity of the engagement process (e.g. active participation, inclusion of relevant perspectives, mutual respect, clear and accountable communication, safety, etc.) and the overall impact of involvement of people with lived experience, not only on the research but also the researcher and public research partner.

Applying a trauma-informed intersectional analysis to existing public involvement in health research evaluative frameworks must include additional metrics to help insure inequities are not reproduced in engagement efforts. Adapting from Olena Hankivsky’s *Intersectionality-Based Policy Framework* [65] as well as *SAMSHA’s Guidance for a Trauma-Informed Approach* [47] the following types of questions should be considered when evaluating public involvement in health research (Table 5).

**Table 5:** Evaluation of public involvement in health research

Questions for the research team (i.e. Researchers and Public Research Partners)
1. How did the way in which people with lived experience were involved in the research project help to reduce health inequities (i.e. avoidable and unjust inequalities in health between and within groups of people)?
2. How often were there opportunities to challenge ideas and renegotiate power within the research project team? How were these moments handled?
3. How did the research team work together to actively define, address and ensure emotional, psychological and physical safety for all research team members?
4. Was there a belief in the primacy of the people, families and communities with lived experience, as well as in the resilience of individuals and communities to heal and promote recovery?
5. Was there an understanding that the experience of trauma may be an aspect that brings us all together and helps to level power differences on the research team?
6. Did the research team understand the importance of differences in power and the way in which certain groups of people, historically, have not had the same opportunity to voice their concerns as well as the same choices as other groups of people, and may have received coercive, disrespectful treatment within the healthcare system?
7. Did the research team make sure to address historical and present-day trauma resulting from colonization, patriarchy, racism, heterosexism, ableism and capitalism?
8. Did the research team actively work to dismantle past cultural stereotypes and biases (e.g. based on race, ethnicity, sexual orientation, age, religion, gender-identity, gender expression, geography, etc.)?
9. Did the research team leverage the healing value of traditional cultural connections?
10. Did the research team incorporate policies, protocols, and processes that are responsive to cultural needs?
11. Did the research team have access to cultural and gender responsive support services in case a researcher or public research partner requires additional support due to past experiences of trauma?

## Conclusion

We believe that public involvement in health research can be a truly transformative practice that can lead to improved health outcomes for all. In order to be a more inclusive and meaningful approach that does not reiterate existing health inequities it is important to re-conceptualize patient engagement through a health equity and social justice lens. We recommend doing so by incorporating a trauma-informed intersectional analysis within the development of training, practice and evaluation with regard to public involvement in health research. This will facilitate the disruption of the identity of “patient” and “researcher” by looking at the simultaneous intersections of social categories within the larger context of connected systems and structures of power, as well as the interdependent forms of privilege and oppression shaped by colonialism, racism, homophobia, ableism and patriarchy created. We argue that in troubling and complicating the identity of patient and researcher we can open up spaces and opportunities to resist and renegotiate power within intersubjective relations within the engagement process.

In training researchers and public research partners in the practice of discursive and embodied reflexivity as well as trauma-informed approaches, it will help them to recognize the role of power and the impact of trauma upon health. The ability to recognize and react to spaces where power can be resisted and renegotiated between researchers and public research partners will help to build trust and transparency, in turn impacting the possible outcomes of the engagement activity and the research as a whole, as well as helping to build resiliency. In the end, this framework will lead to more inclusive and meaningful engagement that will lead to better quality and relevant research, in turn meaning better outcomes for everyone. We believe this framework will help to advance both the practice and science of patient engagement.
